# Indoor Air Pollution: An Old Problem with New Challenges

**DOI:** 10.3390/ijerph6112880

**Published:** 2009-11-19

**Authors:** John Spengler, Gary Adamkiewicz

**Affiliations:** Epidemiology & Risk Assessment Program, Department of Environmental Health, Harvard School of Public Health/Exposure, P.O. Box 15677, Landmark 406 West, 401 Park Ave, Boston, MA 02215 USA; E-Mail: gadamkie@hsph.harvard.edu

Hazards in our indoor environments have been recognized since biblical times. The advice in Leviticus 14:33–48 for treating mold infested houses has contemporary meaning in the recent World Health Organization (WHO) document on damp and moldy indoor spaces [1]. In the developed world, faulty combustion, carbon monoxide from coal gas, lead paint, poor ventilation of tenement housing and hospitals have been recognized for decades as unhealthy. Indoor air quality, however, was not appreciated as an important component of public health until the proliferation of sealed buildings, energy conservation programs (urea formaldehyde foam insulation), new products, and the recognition of the health effects of radon, asbestos and latex. Clinical and epidemiologic studies in the late 1980s through the 1990s documented the increase in allergies and asthma in developed countries. Reports out of India, Nepal, and Kenya on the respiratory effects on children and women from cooking and heating with wood, charcoal and dried animal dung began to receive world attention. The 2002 WHO Global Burden of Disease analysis conservatively attributed 2–3% of the world’s DALY (Disability Adjusted Life-Year) losses to biomass and solid fuels used in the developing countries [2]. The risks from these fuels, as highlighted in the WHO report, continue to constitute the most significant threats to health from indoor pollutants. The evidence against exposures to secondhand smoke (SHS) has also accumulated. The first significant document on secondhand smoke was the US Surgeon General’s 1986 report, which 20 years later was updated and re-published [3]. This updated 2006 report unequivocally stated that there are “no safe levels for secondhand smoke exposures”. With the well-documented public health evidence, the civic debate shifted from one that asserted smokers rights to one that assured non-smokers the right to smoke-free indoor environments.

Today concerns about indoor air quality in developed countries have found expression in green building guidelines, smoking bans, and product standards. Nevertheless, indoor problems persist because of faulty construction, complex building systems, deferred maintenance, new formulations of products and a growing recognition that our homes contribute to our body burden of chemicals. Increased time spent indoors and reduced air exchange rates have increased personal exposure to many compounds originating indoors. Residential environments have the potential to be critical sources of chemical exposures through their release from building materials, household furnishings, and a wide range of consumer products. This diversity of exposures mirrors the dramatic rise in the development and production of synthetic chemicals globally since the 1950s [4]. Many of these compounds, despite being known or suspected developmental toxicants or endocrine disruptors, have not been subject to routine toxicity testing. Their chemical properties, including low volatility, can extend their persistence in homes and confound standard approaches to remove residues that may present health risks for years beyond their use. Some key contaminants include: phthalates, used widely in personal care products and vinyl flooring; PCBs, used in electrical equipment, caulking and surface coatings; chlorinated and brominated flame retardants, used in electronics, furniture and textiles; pesticides, used for pest control in agriculture and the built environment; and parabens, used to preserve products like lotions and sunscreen [5–10]. In the face of these daunting population-level risks, few studies have focused on understanding key exposure pathways within the home and the health risks associated with complex chemical mixtures that may also be influenced by dietary and occupational exposures. The lack of strict labeling regulation for many constituents of household products also limits an individual’s options to control exposure through consumer choices.

We live in an ever more complicated and connected world. A sustainable path for the built environment will depend on our wise use of resources as well as the protection of human health. Energy consumed to build and operate buildings is an obvious component of greenhouse gas reduction strategies. Ambitious energy conservation programs should not lose sight of the lessons learned 40 years ago when sealing buildings with reduced ventilation led to sick building syndrome, combustion source problems and mold with associated health and productivity losses. A warmer atmosphere has already brought increasing weather variability with increased frequency of extreme precipitation and hot spells. For non-air-conditioned homes, warmer nighttime temperatures and prolonged heat events are likely to alter indoor comfort due to the retained thermal mass of the structures, inducing heat stress. Increased humidity and excessive precipitation will lead to dampness in indoor environments and may exacerbate problems with mold. With insight from past indoor air lessons and precautionary use of untested synthetic chemicals, our indoor environments still to be built might offer climate adapted sanctuaries that promote health and reduce carbon emissions.

We are encouraged by recent WHO efforts to focus attention on issues related to housing and health, as well as the U.S. Surgeon General’s recent Call-to-Action on Healthy Homes [11]. We recognize, however, the significant challenges ahead in addressing climate change through energy conservation measures while providing safe, affordable and healthy housing in many settings. In the developing world, the disproportionate use of carbon-intensive fuels in inefficient and polluting cook stoves provides opportunities for carbon reductions with substantial health co-benefits.
